# Functional and Structural Changes in the Corticospinal Tract of Streptozotocin-Induced Diabetic Rats

**DOI:** 10.3390/ijms221810123

**Published:** 2021-09-19

**Authors:** Ken Muramatsu, Satoshi Shimo, Toru Tamaki, Masako Ikutomo, Masatoshi Niwa

**Affiliations:** 1 Department of Physical Therapy, Kyorin University, 5-4-1 Simorenzyaku, Mitaka, Tokyo 181-8612, Japan; 2 Department of Occupational Therapy, Health Science University, 7187 Kodachi, Fujikawaguchiko, Yamanashi 401-0380, Japan; sshimo@kenkoudai.ac.jp; 3 Department of Physical Therapy, Health Science University, 7187 Kodachi, Fujikawaguchiko, Yamanashi 401-0380, Japan; toru-tamaki@kenkoudai.ac.jp; 4 Department of Physical Therapy, University of Tokyo Health Sciences, 4-11 Ochiai, Tama, Tokyo 206-0003, Japan; m-ikutomo@u-ths.ac.jp; 5 Department of Occupational Therapy, Kyorin University, 5-4-1 Simorenzyaku, Mitaka, Tokyo 181-8612, Japan; mt-niwa@ks.kyorin-u.ac.jp

**Keywords:** diabetes, corticospinal tract, diabetic neuropathy

## Abstract

This study aimed to reveal functional and morphological changes in the corticospinal tract, a pathway shown to be susceptible to diabetes. Type 1 diabetes was induced in 13-week-old male Wistar rats administered streptozotocin. Twenty-three weeks after streptozotocin injection, diabetic animals and age-matched control animals were used to demonstrate the conduction velocity of the corticospinal tract. Other animals were used for morphometric analyses of the base of the dorsal funiculus of the corticospinal tract in the spinal cord using both optical and electron microscopy. The conduction velocity of the corticospinal tract decreased in the lumbar spinal cord in the diabetic animal, although it did not decrease in the cervical spinal cord. Furthermore, atrophy of the fibers of the base of the dorsal funiculus was observed along their entire length, with an increase in the g-ratio in the lumbar spinal cord in the diabetic animal. This study indicates that the corticospinal tract fibers projecting to the lumbar spinal cord experience a decrease in conduction velocity at the lumbar spinal cord of these axons in diabetic animals, likely caused by a combination of axonal atrophy and an increased g-ratio due to thinning of the myelin sheath.

## 1. Introduction

Diabetes patients with neuropathy have shown several motor dysfunctions, including an increased risk of falling, altered gait and balance, and increased body sway [1,2,3,4]. These motor dysfunctions were previously believed to be caused by a combination of sensory neuropathy and muscle wasting [5]. However, recent studies have revealed that motor system dysfunction, including the cerebral cortex and spinal cord, can also cause motor dysfunction in these patients [6,7].

We have previously reported disrupted axonal conduction and reductions in the cortical hindlimb motor area of type 1 diabetic rats [8]. Moreover, axonal disruption of the corticospinal tract (CST) is prominent in the long axons that project to the lumbar spinal segment [8]. A previous study reported poor synaptic transmission between the motor cortex and the lumbar spinal motor neurons of short-term type 1 diabetic rats [9], while a clinical study reported a decrease in the volume and conduction velocity of the CST in both type 1 and type 2 diabetes patients [10,11,12,13,14,15]. Understanding the nature of diabetes-related CST injuries is important for revealing the pathophysiology of motor dysfunction in diabetic patients since this tract plays an essential role in controlling voluntary movements [6,16]. However, it is unclear what kind of pathological changes occur in CST axons. Therefore, this study investigated the functional and morphological changes in the long axons of the CST that originate from the hindlimb cortical motor area and connect to the lumbosacral segment.

## 2. Results

### 2.1. Model Animals

Diabetic rats exhibited low body weights and blood glucose levels of approximately 400 mg/dL (464.5 ± 43.0 mg/dL on average, (*p* < 0.0001) vs. 23WC: 149.7 ± 15.0 mg/dL) within 2–3 days of streptozotocin (STZ) injection and throughout the study. The control and diabetic animal groups had similar body weights before the STZ or saline injections. However, the body weights of the control group at 23 weeks after sham diabetes induction (23WC) increased to 449.7 ± 18.8 g on average, whereas the body weight of diabetic animals at 23 weeks after diabetes induction (23WD) was 280.4 ± 6.9 g on the average (*p* < 0.0001, vs. 23WC). The body weights and blood glucose levels of the animals used in each experiment are summarized in Table 1 and Table 2.

### 2.2. Conduction Velocities of the CST and Motor Nerve

**Figure 1:**
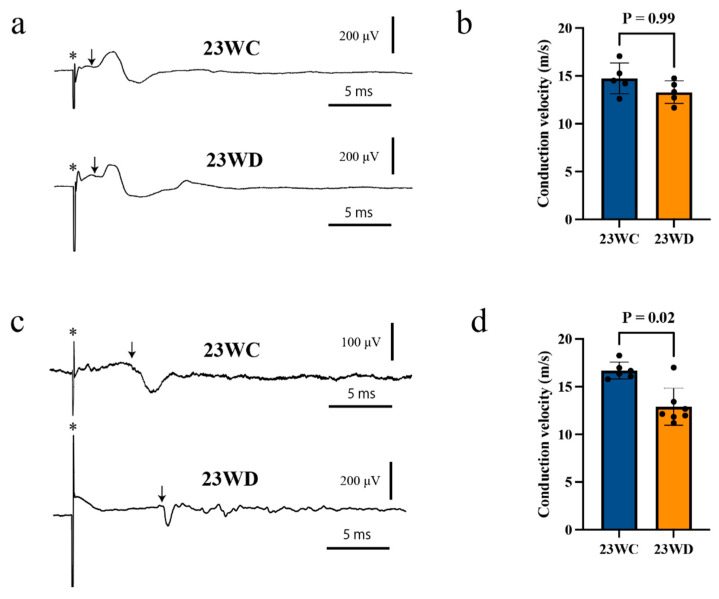
Typical evoked antidromic field potentials of corticospinal tract (CST) neurons. (**a**) Typical hindlimb-area antidromic potentials evoked by C3-segment stimulation in 23WC and 23WD animals. (**b**) There was no significant difference in conduction velocity between diabetic animals and control animals. On the other hand, 23WD showed long-lasting potentials. (**c**) Typical hindlimb-area antidromic potentials evoked by L1-segment stimulation in 23WC and 23WD animals. (**d**) The diabetic animals exhibited decreased conduction velocities and long-lasting multiphasic potentials. Asterisks indicate stimulus artifacts, and arrows indicate each action potential’s first negative deflection. Abbreviations: 23WC, control animals at 23 weeks after sham diabetes induction; 23WD, diabetic animals at 23 weeks after diabetes induction.

**Figure 2:**
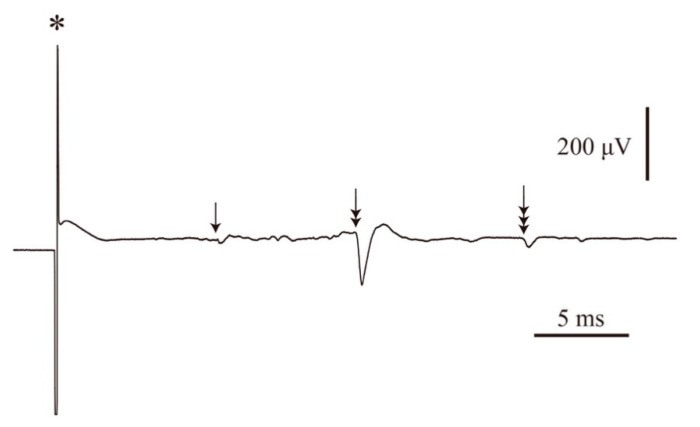
Multiple field potentials of the corticospinal tract (CST) with different conduction velocities. We occasionally observed multiple field potentials in CST neurons with different conduction velocities when applying supra-maximal stimulation to the spinal cord. In this case, the potential with the shortest latency (arrow) is followed by potential with a long latency (double arrow) and then by potential with an extremely long latency (triple arrow). In this case, the conduction velocity of the potential with the longest latency was about 3 m/s. Asterisks indicate stimulus artifacts.

The antidromic field potential of the CST neurons was recorded from the hindlimb cortical motor area. The conduction velocity was then calculated from the shortest latency of the antidromic field potentials, which did not differ between 23WC and 23WD when stimulating the CST at the cervical spinal level (Figure 1a,b, Table 1). However, 23WD showed long-lasting potentials that followed the first sharp potential and frequently merged with the synaptic potential of CST neurons. Therefore, a quantitative analysis of these late potentials was not performed. Meanwhile, the latency for the antidromic field potential of CST neurons of 23WD was prolonged when stimulating the lumbar spinal level. The conduction velocity decreased to 77.2% of 23WC (*p* < 0.05, Figure 1c,d, Table 1). The potentials that followed the first sharp potentials were longer than those with cervical stimulation. Multiple antidromic field potentials with different conduction velocities were recorded in a few cases when stimulating the spinal cord at supra-maximal strength. Figure 2 shows a typical record in which the shortest latency potential is followed by an exceedingly long latency potential. The potentials with the longest latencies sometimes reached 15–20 ms, corresponding to conduction velocities of 2–3 m/s. These data were not included in the analysis because they were unstable, and the extent of the stimulating current effect could not be estimated. Lastly, the motor nerve conduction velocities (MNCVs) were significantly decreased in 23WD (70.9% of 23WC, *p* < 0.001).

### 2.3. Morphological Analysis of Tract Fibers at the Base of the Dorsal Funiculus

**Figure 3:**
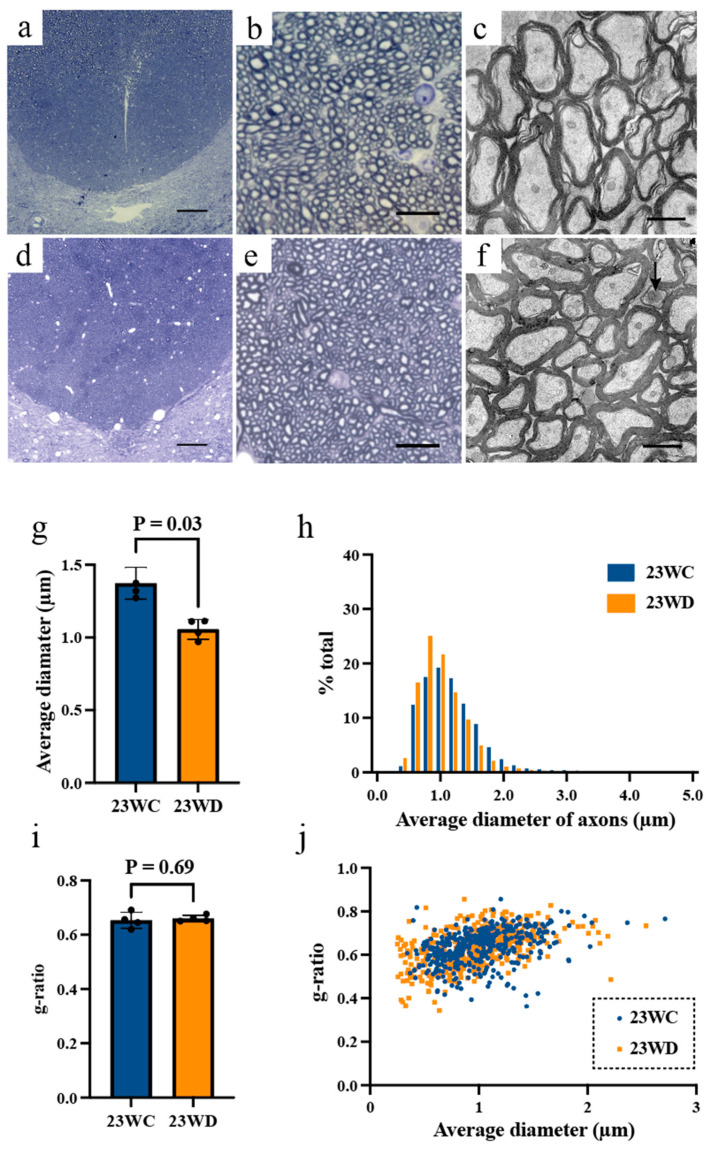
(**a**,**b**) Optical micrographs of the BDF in the cervical spinal cord (**a**) and BDF fibers (**b**) in 23WC. (**c**) Electron micrographs of BDF fibers in 23WC. (**d**,**e**) Optical micrographs of the BDF of the cervical spinal cord (**d**) and BDF fibers (**e**) in 23WD. Evidently, the BDF fibers are thinner in 23WD than 23WC. (**f**) Electron micrographs of BDF fibers in 23WD. The arrow indicates a degenerating axon. No changes in the myelin sheath were observed, and degenerating axons were only rarely observed. The scale bar indicates 100 μm (**a**,**d**), 10 μm (**b**,**e**), and 1 μm (**c**,**f**). (**g**) Average diameter of BDF axons. The size of axons was significantly smaller in 23WD (*p* < 0.05, Mann-Whitney U test). (**h**) Size distribution of BDF axons in the cervical spinal cord in 23WC (n = 12,256 axons) and 23WD (n = 10,673 axons). The Kolmogorov–Smirnov test revealed a significant (*p* < 0.0001) between-group difference. (**i**) g-ratio of BDF fibers. There was no significant difference between the two groups (*p* < 0.05, Mann–Whitney U test). (**j**) The relationship between axonal diameter and the g-ratio in 23WC (n = 420 fibers) and 23WD (n = 431 fibers). There was no difference between the two groups. Abbreviations: BDF, base of the dorsal funiculus; 23WC, control animals at 23 weeks after sham diabetes induction; 23WD, diabetic animals at 23 weeks after diabetes induction.

**Figure 4:**
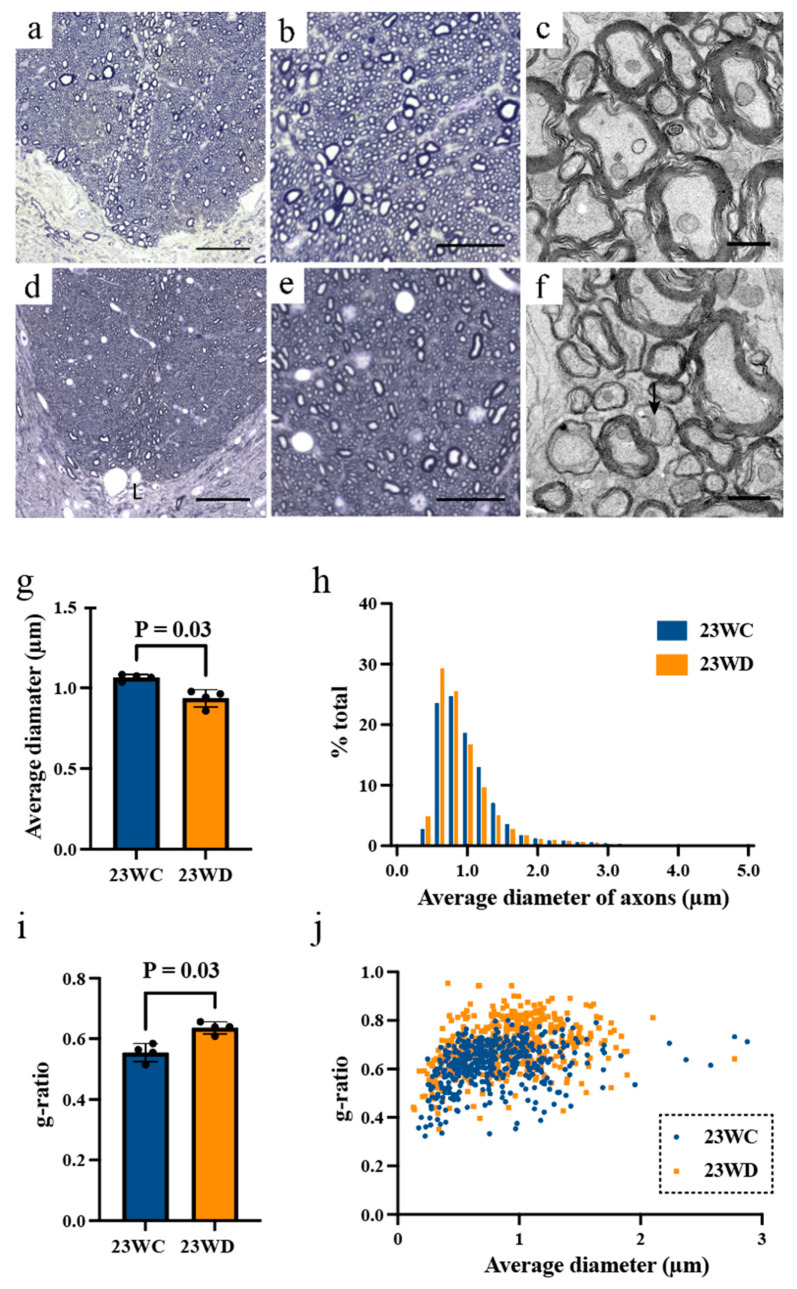
(**a**,**b**) Optical micrographs of the BDF of the lumbar spinal cord (**a**) and BDF fibers (**b**) in 23WC. (**c**) Electron micrographs of BDF fibers in 23WC. (**d**,**e**) Optical micrographs of the bottom of the dorsal column of the cervical spinal cord (**d**) and BDF fibers (**e**) in 23WD. (**f**) Electron micrographs of BDF fibers in 23WD. The arrow indicates a demyelinating axon. The scale bar indicates 100 μm (**a**,**d**), 10 μm (**b**,**e**), and 1 μm (**c**,**f**). (**g**) Average diameter of BDF axons. The axons were significantly smaller in 23WD (*p* < 0.05, Mann–Whitney U test). (**h**) Size distribution of BDF axons in the lumbar cord in 23WC (n = 10,220 axons) and 23WD (n = 9630 axons). The Kolmogorov–Smirnov test revealed a significant (*p* < 0.0001) between-group difference. (**i**) g-ratio of BDF fibers. In 23WD, the g-ratio was significantly higher than in 23WC (*p* < 0.05, Mann–Whitney U test). (**j**) The relationship between axonal diameter and the g-ratio in 23WC (n = 439 fibers) and 23WD (n = 438 fibers). In 23WD, the g-ratio of some BDF fibers increased independent of axonal diameter. Abbreviations: BDF, base of the dorsal funiculus; 23WC, control animals at 23 weeks after sham diabetes induction; 23WD, diabetic animals at 23 weeks after diabetes induction.

Detailed figures for each value are summarized in Table 2. Figure 3a,d, and Figure 4a,d show tissue images from the base of the dorsal funiculus (BDF) of the cervical and lumbar spinal cords occupied by CST fibers [17,18]. As shown in Figure 3b,e,g and Figure 4b,e,g, the myelinated fibers in the dorsal funiculus were generally thinner in 23WD, and the average diameter of the BDF axons was smaller than that of 23WC (*p* < 0.05). The histogram shows an increase in the ratio of thin BDF fibers due to axonal atrophy in the 23WD group, and the distribution was different from that in the 23WC group (Figure 3h and Figure 4h, Kolmogorov–Smirnov test, *p* < 0.0001). 

There were some differences between the cervical and lumbar spinal cords in other measures reflecting the size of long tract axons. In the cervical spinal segment, the cross-sectional area and perimeter of the axons showed only a tendency to be decreased in the 23WD group (*p* = 0.06), while, in the lumbar spinal segment, the cross-sectional area and perimeter of axons in the 23WD group were significantly smaller than those of the 23WC group (*p* < 0.05). In addition to axonal atrophy, the myelin thickness of 23WD reduced in both the cervical and lumbar segments compared to 23WC. The g-ratios were similar (approximately 0.65) at the cervical and lumbar segments of 23WC and the cervical segment of 23WD (Figure 3i,j); however, the g-ratio of the lumbar segment of 23WD showed an increased mean value (0.72 ± 0.03, *p* < 0.05, Figure 4i). As shown in Figure 4j, the increase in the g-ratio at the lumbar spinal cord did not occur uniformly in all long tract fibers. Rather, it was caused by a substantial increase in the g-ratio of some fibers.

The electro-microscopic images reflected this trend more clearly. At the cervical spinal cord level, BDF fibers appeared to have preservation of myelinated structures, although thin axons were evident (Figure 3b,c,e,f). On the other hand, BDF fibers of the lumbar spinal cord were thin and had wide fiber spacing in 23WD, with some fibers having thin myelin sheaths (Figure 4b,c,e,f). Structurally disrupted fibers, i.e., demyelinated or degenerated axons, were rarely observed in the cervical and lumbar spinal cord.

## 3. Discussion

The study found that the axons at the lumbar spinal level developed functional disorders, such as a decreased conduction velocity and morphological abnormalities, such as axonal atrophy and an increased g-ratio. These findings are consistent with the results of our previous study, which demonstrated that diabetes-induced cortical motor area reductions predominantly affected the hindlimb and trunk areas [8].

Electro-physiological analysis revealed that the conduction velocity of CST fibers decreased in 23WD. Because antidromic field potentials from the hindlimb motor area were recorded, these alterations reflected the differences in the effects of diabetes at different portions of the CST fibers that connect to the lumbar spinal level. A comparison between the conduction velocities of stimulated CST fibers at the cervical and lumbar levels revealed that the decrease in conduction velocity only occurred at the lumbar level (Figure 1). This is similar to the finding that peripheral motor nerves have a predominantly distal decrease in conduction velocity in diabetes [19]. Conversely, it was previously reported that the conduction velocity of the CST originating from the forelimb motor cortices and projecting to the cervical spinal cord shows a decreased conduction velocity in 23-week diabetic rats [8]. At first glance, these findings seem to contradict one another. However, it is believed that this discrepancy is because the CST fibers originating from the forelimb area stimulate the terminal portion of the fibers, while the CST fibers originating from the hindlimb region stimulate the central portion of the fibers. The conduction velocity of CST axons calculated by the shortest latency of the antidromic field potential should be noted. This reflects the conduction velocity of the fastest, i.e., the largest, CST axons. A previous study showed that the CST contains fibers of various sizes and conduction velocities; therefore, analyzing only the fastest conduction velocity does not provide a complete picture of its reduction in the CST [20]. The inability to present sufficient data to back up this point is a limitation of this study’s methodology. However, as shown in Figure 2, long-latency antidromic field potentials of CST cells with slow conduction velocities in 23WD were occasionally recorded. Based on these observations, it is believed that the decrease in conduction velocity also occurred in smaller CST fibers with a slower conduction velocity because these potentials were only observed by supra-maximal stimulation, and the conduction velocities of these CST fibers included some as low as 2–3 m/s, which is below the minimum velocity of CST fibers in normal rats (approximately 4 m/s) [20]. This decrease in the nerve conduction velocity may significantly affect the arrival time of motor commands from the cortical motor cortex to the spinal cord. Since the effect of a reduced conduction velocity is greater, the longer the distance from the excitation, the more time for motor commands to arrive at the lumbosacral spinal cord, which is farthest from the cortical motor cortex, may be completely different for each CST fiber. This may make it difficult to supply synchronized excitatory synaptic input to hindlimb motor neurons and provide a good explanation for why diabetes-induced cortical motor area reductions predominantly affect the hindlimb and trunk areas [8].

The morphological observations also reflected these physiological explanations. The morphological analysis focused on the alterations at the BDF, where the CST is located. Since CST axons were not identified using a neurotracer, other axons may also have been included in the axons analyzed. However, it has previously been established that CST axons occupy the BDF of rats and fully reflect the morphological features of CST axons [17,18]. Therefore, the fibers at the BDF are referred to as putative CST (p-CST) fibers for convenience in the discussion. In addition, the CST at the cervical spinal cord contains fibers that project to segments other than the lumbar spinal cord. Since these could not be distinguished in this study, it is another limitation of our experimental methodology.

The quantitative morphological analysis of p-CST fibers revealed that atrophy of axons was diffused over the total length of the p-CST but was most evident in the lumbar spinal segment of 23WD. In addition to morphological changes in axons, a decrease in p-CST fibers myelin thickness was also observed in the cervical and lumbar segments of 23WD. It is widely accepted that the g-ratio is a highly reliable ratio for assessing axonal myelination [21]. Furthermore, it is known that the g-ratio of the CST in young rats is approximately 0.65, which is fairly constant throughout the diameter spectrum [17,18]. As shown in Figure 3i,j, the g-ratio of 23WC showed similar values at the cervical and lumbar segments, as well as the cervical segment of 23WD. However, the g-ratio of the lumbar segment of 23WD showed an increased mean value (0.72 ± 0.03, see Figure 4i and Table 2). It is believed that a g-ratio of 0.7 in the CNS does not cause interference with excitation conduction [21]. However, Figure 4j, shows the increase in the lumbar spinal cord g-ratio did not occur uniformly for all p-CST fibers but was caused by a large increase in the g-ratio of over 0.7 in some p-CST fibers. Therefore, p-CST fibers in lumbar spinal segments with significantly increased g-ratios may substantially impair excitatory conduction when coupled with axonal atrophy since it is known that both the axonal diameter and myelin sheath are important for minimizing conduction delays [21]. This observation provides a good explanation for our electrophysiological data, which demonstrated a decrease in the conduction velocity predominantly in the lumbar spinal segment of p-CST fibers in 23WD. However, why the myelin abnormalities of p-CST axons are more prominent in the lumbar spinal segment is unclear. This could be due to the various known interactions between oligodendrocytes and axons that may influence myelination [22]. Another possibility is that CST fibers degenerate secondary to hindlimb motor neuron damage because MNCVs decreased in 23WD. However, this is unlikely, because previous studies have shown motor neuron damage in the same animal model has little effect on alpha motor neurons [23,24]. On the other hand, it has been previously predicted that axonal retraction contributes to reducing cortical motor area [8]. However, functional alterations in p-CST fibers caused by axonal atrophy and demyelination, rather than axonal retraction, seem to account for most of the changes in the motor cortex because the histological analysis showed that axonal retraction was rarely observed.

In conclusion, defects in the corticospinal tract observed in STZ rats are characterized by axonal atrophy and thinning of the myelinated sheaths in peripheral regions, as well as a decreased nerve conduction velocity and an increased conduction velocity variability, probably due to the aforementioned changes. This decreased conduction velocity and increased variability may explain why synchronized synaptic inputs to the spinal cord are prevented, resulting in the failure of excitatory transmission from the cerebral cortex to MNs. However, limitations of this study include the following: first, it did not measure the conduction velocity of axons in a single cell; therefore, it cannot clarify the extent to which the conduction velocity varied. Second, it did not identify CST fibers using neurotracers and others. A method to visualize entire CST fibers without affecting their morphology needs to be investigated. Third, the 3D ultrastructural architecture of unmyelinated axons, axoplasm, or myelin sheaths of myelinated axons was not observed, making it impossible to investigate the changes within the cells. If the conduction velocity of axons in a single cell could be observed and quantify 3D ultrastructure of CST fibers, a new mechanism of pathogenesis could be found, which should be studied in future.

## 4. Materials and Methods

### 4.1. Induction of Experimental Diabetes (Diabetic Animal Model)

Type 1 diabetes was induced in 13-week-old male Wistar rats (*n* = 11), 270–288 g in body weight, via STZ (100 mg/kg in saline, i.p.) administration, and a plasma glucose level of >400 mg/dL was used to confirm a diabetic status. Age-matched control animals (268–277 g body weight) were injected with saline only (*n* = 10). All animals were housed in plastic cages with a flat bottom covered with a soft bedding material. Food and tap water were provided ad libitum. Animals were maintained in a temperature-controlled room with a light/dark cycle of 12:12 h. Twenty-three weeks after STZ injection—determined based on a previous study demonstrating that diabetes induced during this period caused the most obvious CST injury—the diabetic group (23WD, n = 7) and control group (23WC, n = 6) were used to demonstrate the conduction velocity of the CST [8]. The third group was used for ultrastructural and morphometric analyses (each group containing four animals).

### 4.2. Measurement of the Conduction Velocity of CST Fibers and Motor Nerve

23WD and 23WC rats were used to demonstrate the conduction velocity of CST fibers, as previously described [8]. In brief, animals were anesthetized with ketamine hydrochloride (70 mg/kg, i.p.) and xylazine (5 mg/kg, i.p.), then were mounted in a stereotaxic frame and placed on a heating pad. A rectal thermometer was used to confirm a body temperature of 37 °C. Craniotomies were performed to expose the right sensorimotor cortex, and 1.4-MΩ tungsten recording microelectrodes were inserted at the center of the hindlimb motor area. Laminectomies was performed to expose the dorsal surface of the spinal cord at the C3 and L1 segments to stimulate the CST at the spinal level. Subsequently, 40-kΩ tungsten microelectrodes was stereotaxically inserted (Unique Medical, Tokyo, Japan) at the deepest portion of the left dorsal column where the rat CST was located [17].

To record the antidromic potentials of CST neurons, the spinal cord was stimulated with single-pulse, 1-Hz, 200-μs monophasic cathodal pulses from SEN-7103 stimulators (Nihon Kohden, Tokyo, Japan) and SS-04 J isolators (Nihon Kohden, Tokyo, Japan). The maximum stimulation current was 50 μA to avoid stimulation of other pathways by leaked current pathways. In five animals per group, both the cervical and lumbar spinal segments were stimulated while stimulating only the lumbar segment in the reining animals. The stimulating current was gradually increased until antidromic field potentials were recorded. These potentials were amplified (AB-651 J, Nihon Kohden) and digitized at a 20-kHz sample rate (PowerLab, ADInstruments, Dunedin, New Zealand), and blocks of 10 consecutive potentials were averaged (Chart 8, ADInstruments, Dunedin, New Zealand). A potential with a latency that fluctuated between sweeps was frequently observed. However, these potentials were excluded from analyses because they might have resulted from synaptically activated neurons by somatosensory cortex rather than antidromically activated neurons [20]. The distance from the stimulation point to each recording point was determined at the end of each experiment. The latency of potentials was calculated from the onset time of the stimulus artifact to the point of the first deflection of the evoked potential in the averaged recording. The CST conduction velocities were calculated.

Subsequently, we measured the peripheral MNCVs, exposed the right sciatic nerve and sectioned it at the ischiorectal fossa. The nerves on either side of the cut were mounted on a bipolar silver hook electrode and inserted bipolar wire electrodes into the medial gastrocnemius muscle to record evoked electromyograms. The average MNCVs was determined by stimulating nerves with 2-Hz, 100-μs supra-maximal pulse stimuli. We averaged 10 responses and then calculated the MNCVs by subtracting the distal latency from the proximal latency and dividing the result by the inter-electrode distance.

### 4.3. Morphological Analysis

Anesthesia was initially induced with 4% halothane and then subsequently administered pentobarbital (35 mg/kg, i.p.) and transcardially perfused animals with 500 mL of saline containing heparin sodium followed by a fixative solution of 4% paraformaldehyde and 1% glutaraldehyde in a 0.1-M phosphate buffer (pH 7.4) at 4 °C. The spinal cords were removed and placed in the same solution at 4 °C for 24–48 h, as reported previously [25]. The spinal cords were then sectioned transversely into 1-mm blocks, post-fixed with 1% osmium tetroxide in 0.1 M PB, dehydrated in a graded series of ethanol, and routinely embedded in Epon. The Epon-embedded specimens were first cut into 1.0-μm thick sections with an ultramicrotome, mounted on glass slides (Matsunami Glass, Osaka, Japan), and routinely stained with toluidine blue to identify surface tissue areas under a BZ-X710 multifocal microscope (KEYENCE, Osaka, Japan) for examination and photographing of the dorsal column of the spinal cord. Ultrathin sections were then cut at a 70–90 nm thickness adjacent to the thicker Epon sections. These were doubly stained with uranyl acetate and lead citrate and then observed and photographed using a transmission electron microscope (JEM-2100F, JEOL, Tokyo, Japan) at an accelerating voltage of 200 kV. 

Subsequently, the morphology, axonal size, myelin thickness, and g-ratio (the ratio of the inner axonal diameter to the total outer diameter) of the myelinated fibers located 200 μm from the BDF were analyzed, which is occupied by CST fibers in photographs with ImageJ (National Institutes of Health, Bethesda, MD) [17,18]. The sizes of the axons were measured using optical micrographs. An imaginary oval surrounding the fiber or axon was used for the diameter measurements. The myelin thickness and g-ratio were calculated from a random selection of more than 100 fibers from each group using a combination of optical and electron micrographs because the boundaries between adjacent fibers could be obscured. Although there were larger myelinated fibers scattered in the center of the base of the dorsal column, these were fibers of the ascending tract and were therefore excluded from the analysis [26].

### 4.4. Data Analysis

The data are presented as means ± standard deviations. Between-group comparisons were performed using the Mann–Whitney U test. The Kolmogorov–Smirnov 2-sample test was used to determine whether the distributions were significantly different. All analyses were performed using GraphPad Prism 9 software (GraphPad Software, La Jolla, CA, USA). Statistical significance was defined as a *p*-value of < 0.05, with a tendency toward significance defined as 0.05 ≤ *p* < 0.15.

## Figures and Tables

**Table 1:** Body weights, blood glucose levels, and conduction velocities of CST fibers in animals used for electrophysiological experiments.

	23WC (n = 6)	23WD (n = 7)	*p*-Value
Body weight (g)	449.7 ± 16.6	282.0 ± 6.0	<0.0001
Blood sugar (mg/dL)	152.7 ± 16.3	465.4 ± 46.2	<0.0001
Conduction velocity of the CST (m/s)			
Stimulation: Cervical	14.7 ± 1.6	14.3 ± 1.2	0.99
Stimulation: Lumbar	16.7 ± 0. 9	12.9 ± 1.9	0.02
MNCVs (m/s)	56.3 ± 5.7	39.9 ± 8.2	<0.01

Abbreviations: 23WC, control animals at 23 weeks after sham diabetes induction; 23WD, diabetic animals at 23 weeks after diabetes induction; CST, corticospinal tract; MNCV, motor nerve conduction velocity.

**Table 2:** Body weights, blood glucose levels, and morphological data for BDF fibers in animals used for morphometric experiments.

	23WC (n = 4)	23WD (n = 4)	*p*-Value
Body weight (g)	449.8 ± 24.5	277.5 ± 8.2	<0.0001
Blood sugar (mg/dL)	145.3 ± 13.6	463.1 ± 45.3	<0.0001
Average diameter of axon (μm)			
Cervical	1.37 ± 0.11	1.06 ± 0.07	0.03
Lumbar	1.07 ± 0.02	0.94 ± 0.05	0.03
Cross-sectional area of axon (μm^2^)			
Cervical	1.12 ± 0.20	0.90 ± 0.09	0.06
Lumbar	1.04 ± 0.04	0.78 ± 0.11	0.03
Perimeter of axon (μm)			
Cervical	4.38 ± 0.51	3.78 ± 0.26	0.06
Lumbar	3.96 ± 0.20	3.31 ± 0.28	0.03
G-ratio			
Cervical	0.65 ± 0.03	0.66 ± 0.01	0.69
Lumbar	0.62 ± 0.05	0.72 ± 0.03	0.03
Myelin thickness (μm)			
Cervical	0.54 ± 0.05	0.38 ± 0.01	0.03
Lumbar	0.42 ± 0.03	0.33 ± 0.04	0.03

Abbreviations: BDF, base of the dorsal funiculus; 23WC, control animals at 23 weeks after sham diabetes induction; 23WD, diabetic animals at 23 weeks after diabetes induction; CST, corticospinal tract.
